# Dipyridinium diaqua­bis­(pyrazole-3,5-di­carboxyl­ato-κ^2^ *N*,*O*)cuprate(II) dihydrate

**DOI:** 10.1107/S1600536812046508

**Published:** 2012-11-17

**Authors:** Youtao Si

**Affiliations:** a State Key Laboratory Breeding Base of Humid Subtropical Mountain Ecology, College of Geographical Sciences, Fujian Normal University, Fuzhou 350007, People’s Republic of China; b Université Européenne de Bretagne, Université de Bretagne Occidentale, CS 93837, 29238 Brest Cedex 3, France

## Abstract

In the mononuclear title salt, (C_5_H_6_N)_2_[Cu(C_5_H_2_N_2_O_4_)_2_(H_2_O)_2_]·2H_2_O, the Cu^II^ ion is located on an inversion centre and is coordinated by two chelating pyrazole-3,5-dicarboxyl­ate anions and two water mol­ecules, forming a Jahn–Teller-distorted CuN_2_O_4_ octa­hedron. O—H⋯O and N—H⋯O hydrogen bonds are formed between water mol­ecules, complex anions and the pyridine counter-cations, leading to the formation of layers parallel to (100). The layers are held together by weak C—H⋯O hydrogen bonds.

## Related literature

For more information on ligands derived from pyrazole-3,5-dicarb­oxy­lic acid, see: King *et al.* (2004 ▶). For the bond-valence method, see: Brown (2002 ▶).
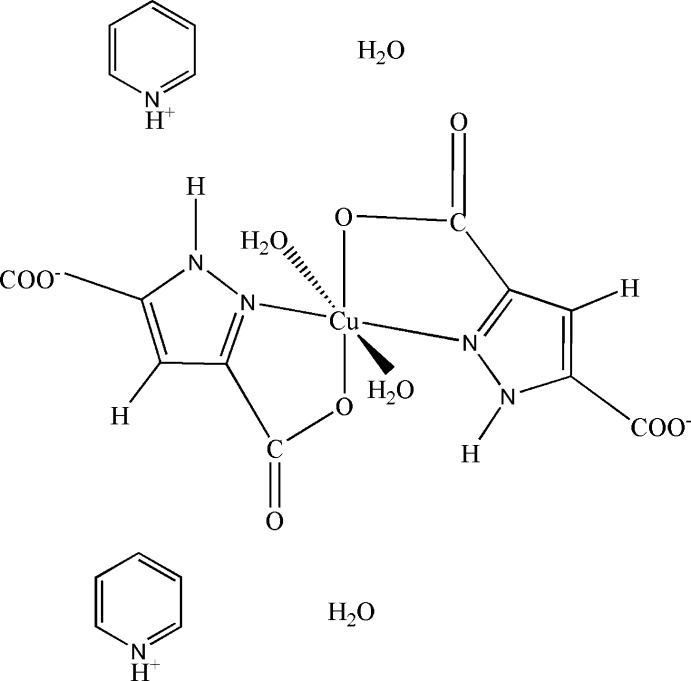


## Experimental

### 

#### Crystal data

(C_5_H_6_N)_2_[Cu(C_5_H_2_N_2_O_4_)_2_(H_2_O)_2_]·2H_2_O
*M* *_r_* = 603.99
Monoclinic, 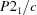

*a* = 9.3531 (4) Å
*b* = 7.3521 (1) Å
*c* = 17.9903 (7) Å
β = 95.600 (2)°
*V* = 1231.20 (7) Å^3^
*Z* = 2
Mo *K*α radiation
μ = 0.96 mm^−1^
*T* = 273 K
0.34 × 0.18 × 0.06 mm

#### Data collection

Bruker SMART CCD diffractometer
Absorption correction: multi-scan (*SADABS*; Sheldrick, 1996 ▶) *T*_min_ = 0.735, *T*_max_ = 0.944
3386 measured reflections
2106 independent reflections
1802 reflections with *I* > 2σ(*I*)
*R*_int_ = 0.038

#### Refinement

*R*[*F* ^2^ > 2σ(*F* ^2^)] = 0.075
*wR*(*F* ^2^) = 0.154
*S* = 1.26
2106 reflections
193 parameters
5 restraints
H atoms treated by a mixture of independent and constrained refinement
Δρ_max_ = 0.50 e Å^−3^
Δρ_min_ = −0.41 e Å^−3^

### 

Data collection: *SMART* (Siemens, 1998 ▶); cell refinement: *SAINT* (Siemens, 1998 ▶); data reduction: *SAINT*; program(s) used to solve structure: *SHELXS97* (Sheldrick, 2008 ▶); program(s) used to refine structure: *SHELXL97* (Sheldrick, 2008 ▶); molecular graphics: *WinGX* (Farrugia, 2012 ▶); software used to prepare material for publication: *publCIF* (Westrip, 2010 ▶).

## Supplementary Material

Crystal structure: contains datablock(s) I, global. DOI: 10.1107/S1600536812046508/wm2698sup1.cif

Structure factors: contains datablock(s) I. DOI: 10.1107/S1600536812046508/wm2698Isup2.hkl

Supplementary material file. DOI: 10.1107/S1600536812046508/wm2698Isup4.cdx

Additional supplementary materials:  crystallographic information; 3D view; checkCIF report

## Figures and Tables

**Table:** 

Cu1—O4	1.959 (4)
Cu1—N2	2.006 (4)
Cu1—O5	2.539 (5)

**Table:** 

O4—Cu1—N2	81.99 (16)
O4—Cu1—O5	90.92 (17)
N2—Cu1—O5	86.97 (17)

**Table 2:** Hydrogen-bond geometry (Å, °)

*D*—H⋯*A*	*D*—H	H⋯*A*	*D*⋯*A*	*D*—H⋯*A*
N1—H1⋯O6^i^	0.86	1.99	2.783 (6)	154
O5—H3*A*⋯O3^ii^	0.81 (4)	1.95 (4)	2.764 (6)	175 (8)
O5—H3*B*⋯O2^iii^	0.82 (2)	2.04 (3)	2.845 (6)	170 (7)
O6—H4*A*⋯O4	0.82 (5)	1.96 (5)	2.735 (7)	159 (7)
O6—H4*B*⋯O1^ii^	0.82 (6)	2.01 (6)	2.801 (6)	162 (7)
N3—H5⋯O1^iv^	0.87 (6)	1.81 (6)	2.665 (7)	171 (6)
C6—H6⋯O5^v^	0.93	2.55	3.247 (9)	132
C8—H8⋯O3^vi^	0.93	2.36	3.211 (8)	151
C10—H10⋯O2^vii^	0.93	2.58	3.231 (8)	128

Symmetry codes: (i) 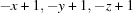; (ii) 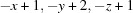; (iii) 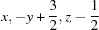; (iv) 
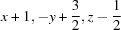
; (v) 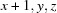; (vi) 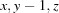; (vii) 
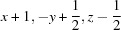
.

**Fig. 1.**
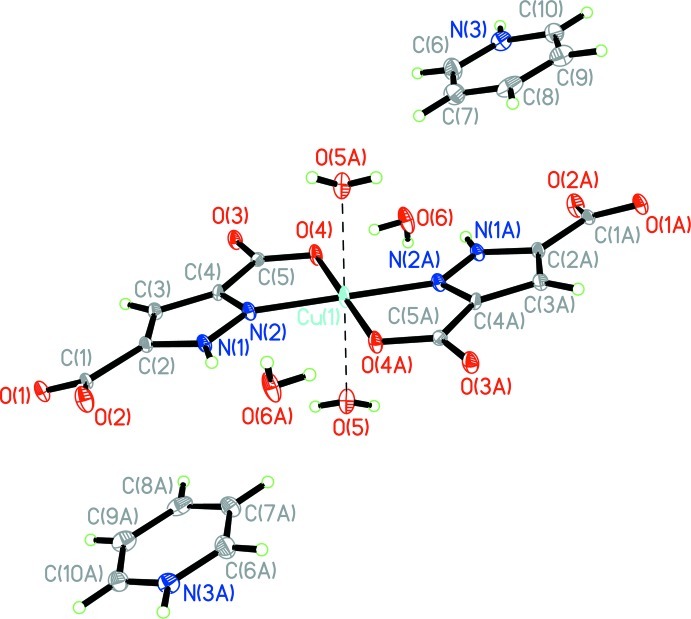
The molecular structure of the title compound, with atom labels and 20% probability displacement ellipsoids for all non-H atoms. [Symmetry code A: -x+1, -y+1, -z+1.]

**Fig. 2.**
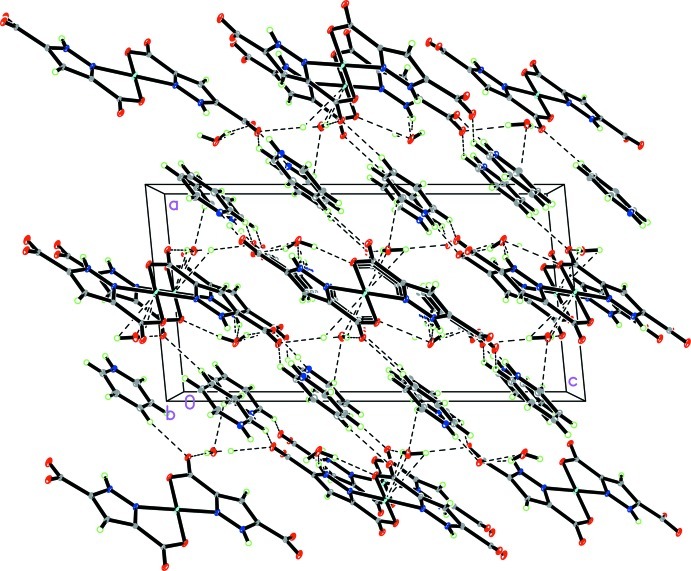
The packing diagram of the title compound, viewed down the b axis. Hydrogen bonding interactions are gived as dashed lines.

## supplementary crystallographic information

### Comment

Pyrazole-3,5-dicarboxylic acid (H_3_dcp) is a versatile ligand with six potential coordinating sites, namely two N atoms of the pyrazole ring and four O atoms of the carboxyl groups. Together with π—π interactions between neighbouring pyrazole rings, the coordination mode of H_3_dcp can lead to different possibilities for creating supramolecular structures (King *et al.*, 2004). When trying to synthesize a copper-containing coordination compound involving H_3_dcp, the title compound, (C_5_H_6_N)^+^_2_[Cu(C_5_H_2_N_2_O_4_)_2_(H_2_O)_2_]^2-^^.^2H_2_O, was obtained.

The Cu^II^ ion sits on an inversion center. One fully deprotonated pyrazole-3,5-dicarboxylic acid, one coordinating water molecule, one lattice water molecule and one pyridinium cation are also present in the asymmetric unit. The other half of the metal-containing moiety is generated by the inversion centre. Each pyrazole-3,5-dicarboxylate anion chelates the metal by one N atom and one O atom in the equatorial plane of an octahedron whereas the axial ligands are provided from water molecules at considerably longer distances (Fig. 1), in agreement with the tetragonal Jahn-Teller distortion (Table 1).

The BVS calculation (Brown, 2002) of the Cu ion gave a value of 1.88 valence units, which indicates that the Cu ion is divalent. Because pyrazole-3,5-dicarboxylic acid is a rather strong acid, the terminal non-coordinating carboxyl group also loses its proton to make the solvent pyridine molecules protonated. Besides four protonated pyridine molecules, four lattice water molecules are present in one unit cell as solvent molecules.

Classical O—H···O and N—H···O hydrogen bonding occurs between water molecules, pyridinium cations and complex anions to form a layer in (100), in which N1, N3, O5, O6 act as donor atoms, and O1, O2, O3, O4, O6 are acceptors (Table 2). The packing of adjacent layers along [100] is accomplished through non-classical weak C—H···O contacts, with the donor belonging to pyridine and acceptor being O atoms of the non-coordinating carboxylate groups.

### Experimental

0.5 mmol H_3_dcp and 0.05 mmol CuCl were mixted in 10 ml H_2_O to give a suspension. After addition of 0.5 ml pyridine, the suspension turned to solution, which was then stirred for 4 h and filtered. After standing in ambient conditions for about 3 days, the filtrate yielded blue crystals suitable for X-ray diffraction.

### Refinement

The H atoms on N3, O5 and O6 were found in the difference electron density map, and the corresponding N—H bond length was set at 0.86 Å, the O—H bond length at 0.82 Å. Other H atoms were placed at idealized positions and allowed to ride on their parent atoms, with C—H and N—H bonds being 0.930 Å and 0.86 Å, respectively. For all H atoms, *U*_iso_(H)=1.2*U*_eq_(C, N or O).

### Crystal data

**Table:**

(C_5_H_6_N)_2_[Cu(C_5_H_2_N_2_O_4_)_2_(H_2_O)_2_]·2H_2_O	*F*(000) = 622
*M**_r_* = 603.99	*D*_x_ = 1.629 Mg m^−^^3^
Monoclinic, *P*2_1_/*c*	Mo *K*α radiation, λ = 0.71073 Å
Hall symbol: -P 2ybc	Cell parameters from 1917 reflections
*a* = 9.3531 (4) Å	θ = 2.2–25.0°
*b* = 7.3521 (1) Å	µ = 0.96 mm^−^^1^
*c* = 17.9903 (7) Å	*T* = 273 K
β = 95.600 (2)°	Prism, blue
*V* = 1231.20 (7) Å^3^	0.34 × 0.18 × 0.06 mm
*Z* = 2	

### Data collection

**Table:**

Bruker SMART CCD diffractometer	2106 independent reflections
Radiation source: fine-focus sealed tube	1802 reflections with *I* > 2σ(*I*)
Graphite monochromator	*R*_int_ = 0.038
phi and ω scans	θ_max_ = 25.0°, θ_min_ = 2.2°
Absorption correction: multi-scan (*SADABS*; Sheldrick, 1996)	*h* = −11→9
*T*_min_ = 0.735, *T*_max_ = 0.944	*k* = −8→7
3386 measured reflections	*l* = −21→10

### Refinement

**Table:**

Refinement on *F*^2^	Primary atom site location: structure-invariant direct methods
Least-squares matrix: full	Secondary atom site location: difference Fourier map
*R*[*F*^2^ > 2σ(*F*^2^)] = 0.075	Hydrogen site location: inferred from neighbouring sites
*wR*(*F*^2^) = 0.154	H atoms treated by a mixture of independent and constrained refinement
*S* = 1.26	*w* = 1/[σ^2^(*F*_o_^2^) + (0.0117*P*)^2^ + 6.7689*P*] where *P* = (*F*_o_^2^ + 2*F*_c_^2^)/3
2106 reflections	(Δ/σ)_max_ < 0.001
193 parameters	Δρ_max_ = 0.50 e Å^−^^3^
5 restraints	Δρ_min_ = −0.41 e Å^−^^3^

### Special details

**Table:**

Geometry. All e.s.d.'s (except the e.s.d. in the dihedral angle between two l.s. planes) are estimated using the full covariance matrix. The cell e.s.d.'s are taken into account individually in the estimation of e.s.d.'s in distances, angles and torsion angles; correlations between e.s.d.'s in cell parameters are only used when they are defined by crystal symmetry. An approximate (isotropic) treatment of cell e.s.d.'s is used for estimating e.s.d.'s involving l.s. planes.
Refinement. Refinement of *F*^2^ against ALL reflections. The weighted *R*-factor *wR* and goodness of fit *S* are based on *F*^2^, conventional *R*-factors *R* are based on *F*, with *F* set to zero for negative *F*^2^. The threshold expression of *F*^2^ > σ(*F*^2^) is used only for calculating *R*-factors(gt) *etc*. and is not relevant to the choice of reflections for refinement. *R*-factors based on *F*^2^ are statistically about twice as large as those based on *F*, and *R*- factors based on ALL data will be even larger.

### Fractional atomic coordinates and isotropic or equivalent isotropic displacement parameters (Å^2^)

**Table:**

	*x*	*y*	*z*	*U*_iso_*/*U*_eq_	
Cu1	0.5000	0.5000	0.5000	0.0307 (3)	
O1	0.3159 (5)	1.0520 (5)	0.7574 (2)	0.0340 (10)	
O3	0.7070 (4)	0.9725 (6)	0.5173 (2)	0.0334 (10)	
N1	0.3911 (5)	0.6681 (6)	0.6461 (2)	0.0253 (11)	
H1	0.3367	0.5826	0.6599	0.030*	
O4	0.6331 (4)	0.6957 (5)	0.4799 (2)	0.0303 (10)	
N2	0.4725 (5)	0.6559 (6)	0.5890 (2)	0.0250 (10)	
C2	0.4060 (6)	0.8331 (8)	0.6791 (3)	0.0238 (12)	
O2	0.2622 (5)	0.7604 (6)	0.7754 (2)	0.0405 (11)	
C1	0.3205 (6)	0.8838 (8)	0.7425 (3)	0.0265 (13)	
C4	0.5402 (6)	0.8162 (7)	0.5856 (3)	0.0220 (12)	
O5	0.2915 (6)	0.6726 (6)	0.4319 (3)	0.0425 (11)	
H3A	0.290 (8)	0.779 (4)	0.444 (4)	0.051*	
H3B	0.287 (8)	0.679 (10)	0.3864 (12)	0.051*	
C5	0.6349 (6)	0.8345 (7)	0.5240 (3)	0.0221 (12)	
C3	0.5020 (6)	0.9310 (8)	0.6420 (3)	0.0270 (13)	
H2	0.5345	1.0487	0.6523	0.032*	
O6	0.7456 (6)	0.6665 (6)	0.3457 (3)	0.0482 (13)	
H4A	0.719 (8)	0.703 (10)	0.385 (2)	0.058*	
H4B	0.709 (8)	0.739 (9)	0.315 (3)	0.058*	
N3	1.1229 (6)	0.2882 (8)	0.3354 (3)	0.0381 (13)	
H5	1.185 (6)	0.350 (8)	0.313 (3)	0.046*	
C9	1.0145 (7)	0.0054 (11)	0.3514 (4)	0.0479 (17)	
H9	1.0028	−0.1166	0.3385	0.057*	
C10	1.1080 (7)	0.1140 (9)	0.3174 (4)	0.0408 (16)	
H10	1.1615	0.0649	0.2814	0.049*	
C7	0.9545 (8)	0.2621 (13)	0.4227 (4)	0.056 (2)	
H7	0.9016	0.3144	0.4583	0.067*	
C8	0.9389 (7)	0.0817 (11)	0.4047 (4)	0.0496 (19)	
H8	0.8760	0.0102	0.4291	0.059*	
C6	1.0501 (8)	0.3639 (11)	0.3870 (4)	0.0512 (19)	
H6	1.0637	0.4862	0.3989	0.061*	

### Atomic displacement parameters (Å^2^)

**Table:**

	*U*^11^	*U*^22^	*U*^33^	*U*^12^	*U*^13^	*U*^23^
Cu1	0.0437 (6)	0.0249 (5)	0.0268 (5)	−0.0106 (5)	0.0198 (4)	−0.0103 (5)
O1	0.042 (3)	0.029 (2)	0.033 (2)	0.0026 (19)	0.018 (2)	−0.0069 (18)
O3	0.038 (2)	0.031 (2)	0.034 (2)	−0.009 (2)	0.0175 (19)	−0.0081 (19)
N1	0.030 (3)	0.024 (2)	0.024 (2)	−0.001 (2)	0.013 (2)	−0.002 (2)
O4	0.040 (2)	0.027 (2)	0.027 (2)	−0.0115 (18)	0.0212 (19)	−0.0082 (17)
N2	0.031 (3)	0.024 (2)	0.021 (2)	−0.001 (2)	0.012 (2)	−0.001 (2)
C2	0.027 (3)	0.027 (3)	0.018 (3)	0.002 (2)	0.004 (2)	−0.003 (2)
O2	0.059 (3)	0.034 (2)	0.032 (2)	−0.004 (2)	0.024 (2)	0.002 (2)
C1	0.027 (3)	0.034 (3)	0.020 (3)	0.008 (3)	0.004 (2)	−0.007 (3)
C4	0.026 (3)	0.021 (3)	0.019 (3)	−0.004 (2)	0.004 (2)	−0.005 (2)
O5	0.068 (3)	0.030 (2)	0.030 (2)	−0.004 (2)	0.006 (2)	−0.002 (2)
C5	0.021 (3)	0.025 (3)	0.021 (3)	−0.001 (2)	0.003 (2)	−0.001 (2)
C3	0.028 (3)	0.025 (3)	0.029 (3)	−0.004 (2)	0.009 (3)	−0.004 (2)
O6	0.078 (4)	0.030 (3)	0.042 (3)	−0.004 (2)	0.037 (3)	−0.005 (2)
N3	0.037 (3)	0.046 (3)	0.033 (3)	−0.009 (3)	0.012 (2)	0.009 (3)
C9	0.045 (4)	0.049 (4)	0.050 (4)	−0.009 (4)	0.002 (3)	0.012 (4)
C10	0.042 (4)	0.046 (4)	0.035 (4)	0.000 (3)	0.008 (3)	0.001 (3)
C7	0.039 (4)	0.092 (6)	0.040 (4)	−0.002 (4)	0.019 (3)	−0.016 (4)
C8	0.037 (4)	0.076 (5)	0.035 (4)	−0.020 (4)	0.000 (3)	0.018 (4)
C6	0.051 (4)	0.051 (4)	0.054 (4)	−0.006 (4)	0.018 (4)	−0.013 (4)

### Geometric parameters (Å, º)

**Table:**

Cu1—O4	1.959 (4)	O5—H3A	0.82 (2)
Cu1—O4^i^	1.959 (4)	O5—H3B	0.82 (2)
Cu1—N2	2.006 (4)	C3—H2	0.9300
Cu1—N2^i^	2.006 (4)	O6—H4A	0.82 (2)
Cu1—O5	2.539 (5)	O6—H4B	0.82 (2)
Cu1—O5^i^	2.539 (5)	N3—C10	1.325 (9)
O1—C1	1.267 (7)	N3—C6	1.325 (8)
O3—C5	1.230 (6)	N3—H5	0.87 (2)
N1—N2	1.341 (6)	C9—C8	1.367 (10)
N1—C2	1.351 (7)	C9—C10	1.371 (9)
N1—H1	0.8600	C9—H9	0.9300
O4—C5	1.292 (6)	C10—H10	0.9300
N2—C4	1.342 (7)	C7—C8	1.369 (11)
C2—C3	1.374 (8)	C7—C6	1.373 (10)
C2—C1	1.502 (7)	C7—H7	0.9300
O2—C1	1.239 (7)	C8—H8	0.9300
C4—C3	1.393 (7)	C6—H6	0.9300
C4—C5	1.491 (7)		
			
O4—Cu1—O4^i^	179.999 (1)	H3A—O5—H3B	102 (7)
O4—Cu1—N2	81.99 (16)	O3—C5—O4	124.5 (5)
O4^i^—Cu1—N2	98.01 (16)	O3—C5—C4	121.2 (5)
O4—Cu1—N2^i^	98.01 (16)	O4—C5—C4	114.3 (5)
O4^i^—Cu1—N2^i^	81.99 (16)	C2—C3—C4	105.2 (5)
N2—Cu1—N2^i^	179.999 (1)	C2—C3—H2	127.4
O4—Cu1—O5	90.92 (17)	C4—C3—H2	127.4
O4^i^—Cu1—O5	89.08 (17)	H4A—O6—H4B	103 (7)
N2—Cu1—O5	86.97 (17)	C10—N3—C6	121.9 (6)
N2^i^—Cu1—O5	93.03 (17)	C10—N3—H5	117 (5)
N2—N1—C2	110.8 (4)	C6—N3—H5	121 (5)
N2—N1—H1	124.6	C8—C9—C10	118.0 (7)
C2—N1—H1	124.6	C8—C9—H9	121.0
C5—O4—Cu1	116.0 (3)	C10—C9—H9	121.0
N1—N2—C4	106.3 (4)	N3—C10—C9	120.7 (7)
N1—N2—Cu1	141.0 (4)	N3—C10—H10	119.6
C4—N2—Cu1	111.6 (3)	C9—C10—H10	119.6
N1—C2—C3	107.6 (5)	C8—C7—C6	118.5 (7)
N1—C2—C1	121.1 (5)	C8—C7—H7	120.8
C3—C2—C1	131.3 (5)	C6—C7—H7	120.8
O2—C1—O1	126.0 (5)	C9—C8—C7	120.8 (7)
O2—C1—C2	118.2 (5)	C9—C8—H8	119.6
O1—C1—C2	115.8 (5)	C7—C8—H8	119.6
N2—C4—C3	110.1 (5)	N3—C6—C7	120.1 (7)
N2—C4—C5	115.5 (4)	N3—C6—H6	120.0
C3—C4—C5	134.4 (5)	C7—C6—H6	120.0
			
N2—Cu1—O4—C5	−5.9 (4)	Cu1—N2—C4—C5	−7.8 (6)
N2^i^—Cu1—O4—C5	174.1 (4)	Cu1—O4—C5—O3	−176.5 (4)
C2—N1—N2—C4	−0.1 (6)	Cu1—O4—C5—C4	3.3 (6)
C2—N1—N2—Cu1	−166.3 (5)	N2—C4—C5—O3	−176.9 (5)
O4—Cu1—N2—N1	173.2 (6)	C3—C4—C5—O3	4.2 (10)
O4^i^—Cu1—N2—N1	−6.8 (6)	N2—C4—C5—O4	3.3 (7)
O4—Cu1—N2—C4	7.4 (4)	C3—C4—C5—O4	−175.6 (6)
O4^i^—Cu1—N2—C4	−172.6 (4)	N1—C2—C3—C4	0.8 (6)
N2—N1—C2—C3	−0.5 (6)	C1—C2—C3—C4	−176.5 (6)
N2—N1—C2—C1	177.2 (5)	N2—C4—C3—C2	−0.9 (7)
N1—C2—C1—O2	16.6 (8)	C5—C4—C3—C2	178.0 (6)
C3—C2—C1—O2	−166.4 (6)	C6—N3—C10—C9	0.7 (11)
N1—C2—C1—O1	−163.7 (5)	C8—C9—C10—N3	−0.8 (10)
C3—C2—C1—O1	13.3 (9)	C10—C9—C8—C7	1.1 (11)
N1—N2—C4—C3	0.6 (6)	C6—C7—C8—C9	−1.3 (11)
Cu1—N2—C4—C3	171.3 (4)	C10—N3—C6—C7	−0.9 (11)
N1—N2—C4—C5	−178.5 (5)	C8—C7—C6—N3	1.1 (12)

Symmetry code: (i) −*x*+1, −*y*+1, −*z*+1.

### Hydrogen-bond geometry (Å, º)

**Table:**

*D*—H···*A*	*D*—H	H···*A*	*D*···*A*	*D*—H···*A*
N1—H1···O6^i^	0.86	1.99	2.783 (6)	154
O5—H3*A*···O3^ii^	0.81 (4)	1.95 (4)	2.764 (6)	175 (8)
O5—H3*B*···O2^iii^	0.82 (2)	2.04 (3)	2.845 (6)	170 (7)
O6—H4*A*···O4	0.82 (5)	1.96 (5)	2.735 (7)	159 (7)
O6—H4*B*···O1^ii^	0.82 (6)	2.01 (6)	2.801 (6)	162 (7)
N3—H5···O1^iv^	0.87 (6)	1.81 (6)	2.665 (7)	171 (6)
C6—H6···O5^v^	0.93	2.55	3.247 (9)	132
C8—H8···O3^vi^	0.93	2.36	3.211 (8)	151
C10—H10···O2^vii^	0.93	2.58	3.231 (8)	128

Symmetry codes: (i) −*x*+1, −*y*+1, −*z*+1; (ii) −*x*+1, −*y*+2, −*z*+1; (iii) *x*, −*y*+3/2, *z*−1/2; (iv) *x*+1, −*y*+3/2, *z*−1/2; (v) *x*+1, *y*, *z*; (vi) *x*, *y*−1, *z*; (vii) *x*+1, −*y*+1/2, *z*−1/2.
